# Development of a blended emergent research training program for clinical nurses (part 1)

**DOI:** 10.1186/s12912-021-00786-x

**Published:** 2022-01-04

**Authors:** Qirong Chen, Zeen Li, Siyuan Tang, Chuyi Zhou, Aimee R. Castro, Shan Jiang, Chongmei Huang, Jinnan Xiao

**Affiliations:** 1 Xiangya School of Nursing, Centreal South University, 172 Tongzipo Road, Changsha, 410013 Hunan China; 2 Ingram School of Nursing, McGill University, 680 Sherbrooke West, Suite 1800, Montreal, QC H3A 2M7 Canada; 3 School of Educational Science, Anhui Normal University, 1 East Beijing Road, Wuhu, 241000 China

**Keywords:** Nursing research competence, Instructional design, Emergent teaching, Blended learning, ADDIE

## Abstract

**Background:**

Nursing research training is important for improving the nursing research competencies of clinical nurses. Rigorous development of such training programs is crucial for ensuring the effectiveness of these research training programs. Therefore, the objectives of this study are: (1) to rigorously develop a blended emergent research training program for clinical nurses based on a needs assessment and related theoretical framework; and (2) to describe and discuss the uses and advantages of the ADDIE model (Analysis, Design, Development, Implementation, Evaluation) in the instructional design and potential benefits of the blended emergent teaching method.

**Methods:**

This intervention development study was conducted in 2017, using a mixed-methods design. A theoretical framework of blended emergent teaching was constructed to provide theoretical guidance for the training program development. Nominal group technique was used to identify learners’ common needs and priorities. The ADDIE model (Analysis, Design, Development, Implementation, Evaluation) was followed to develop the research training program for clinical nurses based on the limitations of current nursing research training programs, the needs of clinical nurses, and the theoretical foundation of blended emergent teaching.

**Results:**

Following the ADDIE model, a blended emergent research training program for clinical nurses to improve nursing research competence was developed based on the needs of clinical nurses and the theoretical framework of blended emergent teaching.

**Conclusions:**

This study indicates that nominal group technique is an effective way to identify learners’ common needs and priorities, and that the ADDIE model is a valuable process model to guide the development of a blended emergent training program. Blended emergent teaching is a promising methodology for improving trainees’ learning initiative and educational outcomes. More empirical studies are needed to further evaluate blended emergent teaching to promote the development of related theories and practice in nursing education.

**Supplementary Information:**

The online version contains supplementary material available at 10.1186/s12912-021-00786-x.

## Background

Nursing research capacity refers to the ability to conduct nursing research activities in a sustainable manner in a specific context, and this concept is normally used at a non-individual level [1]. Nursing research capacity is critical for the development of the nursing discipline, as well as for positive nurse, patient and healthcare system outcomes [2]. Nursing research capacity requires not only individual nursing research competence (NRC), but also contextual factors that exist to support and sustain research activities [3]. Nursing research competence refers to an individual’s ability to conduct nursing research activities (i.e., identify problems and formulate research questions, search and critically review literature, design and implement research, analyze data, and write research reports) [4]. Thus, NRC is crucial for nursing research capacity building, as it is a prerequisite for contextual factors to play a role in nurturing the group/organization/discipline’s overall nursing research capacity [1, 5, 6].

With the development of the nursing discipline and evidence-based nursing practice, NRC is an increasingly important competency for clinical nurses [7, 8]. The nursing discipline and evidence-based nursing practice both require high-quality clinical nursing studies that will generate further clinical nursing knowledge and evidence. As the providers of direct care to patients in hospitals, clinical nurses are crucial for clinical nursing research. Clinical nurses have rich bedside experiences that can inspire numerous research questions that are highly relevant to clinical practice [9]. Furthermore, these nurses have advantageous positions for participant recruitment and data collection. However, to successfully implement evidence-based nursing practice, clinical nurses must be equipped with adequate NRC, which is necessary for the synthesis of evidence [6]. Although contextual supports (e.g, time, resources, funding, supportive institutional culture, etc.) are necessary to successfully conduct high-quality nursing clinical studies and evidence-based nursing practice [10], but NRC of clinical nurses is a prerequisite for contextual supports to play their supportive roles [1].

Low NRC of clinical nurses is a worldwide problem that is hindering the development of the nursing discipline, as well as the implementation of evidence-based nursing practice [6, 11]. Training programs have been recognized as an important method to improve NRC, particularly for clinical nurses who do not always have the opportunity to further their academic education in universities [11–13]. For clinical nurses who have some experience in nursing research, research training could provide them with opportunities to systematically learn or review research knowledge and skills. For clinical nurses with limited research experience, a more comprehensive introduction to research methods could inspire their interest in nursing research [14].

However, there is a lack of effective research training programs for clinical nurses, and related intervention studies are limited. Based on a broad and comprehensive literature search and review, 14 studies that mainly focus on the effectiveness of research training programs for clinical nurses were found [5, 14–17]. They were conducted in China (10 studies), England (2 studies), Denmark (1 study), and Spain (1 study). While they offer an important starting point for the study of research training for clinical nurses, these studies have several limitations. Firstly, the evidence they provide is limited because of the limitations of the study designs (e.g., a lack of control groups, no consideration of contamination, the use of incorrect statistical methods, etc.). None of the studies included a specific description of a theoretical framework to provide the necessary theoretical guidance for the training program development; nor were there specific descriptions of the development processes of the research training programs. Furthermore, no significant, innovative strategies targeting the nursing research competence of clinical nurses could be identified in any of these research training programs [5, 14–17]. These limitations align with the unsatisfactory outcomes in these intervention studies. In sum, there is still a paucity of research that: (1) is focused on the development of innovative research training programs for clinical nurses, and (2) offers convincing empirical studies to evaluate the effectiveness of such research training programs on clinical nurses.

Therefore, the goal of the entire research project was to innovatively develop and evaluate an appropriate, systematic and effective research training program for clinical nurses to improve NRC. This paper illustrates the development of the training program for this research project. The evaluation of this training program is described in a separate intervention evaluation paper [18]. Based on the literature review and a focus group interview of clinical nurses in this project, the limitations of previous nursing research training programs and corresponding solutions are proposed (for further details on these limitations and proposed solutions, see the “*Analysis**”* component of Section 2. Method; Section 3. Results; and Table S1). Guided by the suggested solutions in Table S1, a blended emergent research training program for clinical nurses was proposed as a potentially effective way to improve the NRC of clinical nurses, based on (1) a needs assessment, and (2) the theoretical framework of blended emergent teaching. Blended emergent learning is a combination of emergent teaching and blended learning (see more details under the “*Analysis*” component of Section 3. Results). Emergent teaching is a learner-centered teaching approach in which educators and learners dynamically develop and explore knowledge together [19]. Blended learning is a blend of online learning and face-to-face learning. Blended learning helps to address many of the limitations of learning when it is conducted solely online (e.g., reduced relationship-building) or solely face-to-face (e.g., lack of scheduling flexibility) [20].

### Objectives of this study

The primary objective of this study was to develop a blended emergent research training program for clinical nurses based on a needs assessment and related theoretical framework. The secondary objective was to describe and discuss the use of the ADDIE model (see more details in Section 2. Method) in the instructional design and potential benefits of the blended emergent teaching method on nursing education. (The evaluation of this training program have been described specifically in a separate intervention evaluation paper [18].)

### The context of this study

This project was conducted in China in 2017, where more than 75% of nurses have less than a bachelor’s degree. However, even among university-educated clinical nurses, the majority of these nurses do not receive research education as an essential part of their formal curriculum. Instead, short lectures, conferences, workshops, short-term (3–5 day) training programs, and journal clubs are the most common methods for disseminating research training to clinical nurses. Most of these training programs are non-systematic, time-limited, and teacher-centered [17].

The participants in our research project were clinical nurses with a bachelor’s degree or master’s degree who have some, but limited, knowledge of nursing research. After multiple discussions within the research team, we decided to exclude clinical nurses without a bachelor’s degree, because nurses without university education rarely receive any training or education in nursing research. Thus, they are not equipped with research skills and do not anticipate working in clinical research. Even with this population restriction, most participants received limited formal and systematic research education as part of their university educations. More details can be found in the notes of Table S1 and Table S2.

## Methods

**Fig. 1:**
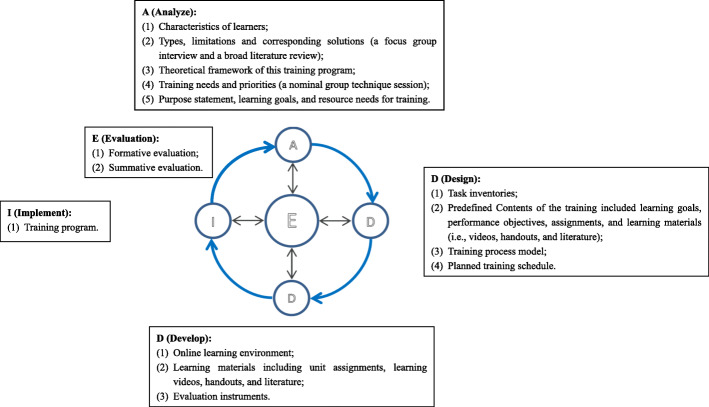
ADDIE model and main elements in each step in this study

The described project was an intervention development study using a mixed-methods design. Both quantitative and qualitative data were collected in this study for intervention development. We used ADDIE (an instructional design paradigm) (Fig. 1) [21] as the process model for training development. ADDIE is appropriate for effectively developing educational products and learning resources [21]. However, this model has received limited attention from nursing educators and nursing education researchers. The ADDIE model includes Analysis, Design, Development, Implementation, and Evaluation stages. Evaluation includes formative evaluation and summative evaluation. The ADDIE model is an iterative process model, whereby a program developer can switch to the Evaluation stage at any time, and then return to any other steps (A, D, D, I) based on the results of the evaluation. Ethical approval of this study was provided by the Institutional Review Board of Behavioral and Nursing Research in the School of Nursing of Central South University (No. 2017033).

### Analysis

The characteristics of learners and other results were analyzed throughout the ADDIE process. A focus group interview of 10 clinical nurses, as well as a broad literature review, were used to identify the common types and limitations of current nursing research training programs and to propose corresponding solutions. The theoretical frameworks of the various training programs identified in the focus group and literature review were analyzed. The focus group participants were all nurses who: (1) provided direct nursing care to patients in hospitals, (2) had a a bachelor’s degree or higher, and (3) had prior experiences in research training and nursing research activities. The nominal group technique, which is a common consensus-building method [22], was used as a needs assessment technique to identify learners’ common training needs and training priorities. Details of the nominal group technique session are described in Fig. S1 and the note of Table S2, respectively [22]. The purpose statement and learning goals of the training were proposed based on the results of the nominal group technique session. All resources needed for training were also analyzed.

### Design

Task inventories were created to organize the learning goals, the performance tasks required to achieve each learning goal, and the prerequisite knowledge and skills for completing each performance task (an example is shown in Fig. S2). These task inventories were critical for appropriately designing the instructions and for helping learners to effectively construct the knowledge and skills required to achieve the learning goals. (More details on Task Inventory can be found in the ADDIE guidance book [21].) The task inventory of every training unit was designed based on the results of the purpose statement and learning goals (Table S3) from the first step (*Analysis*).

**Fig. 2:**
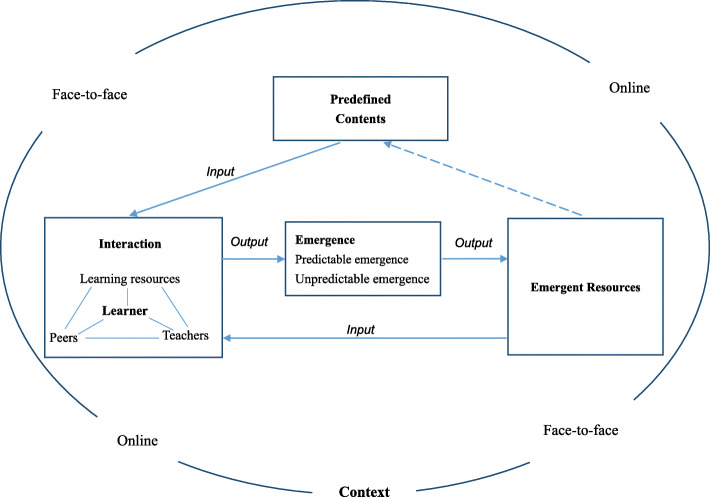
The theoretical framework of blended emergent teaching

In this blended emergent training program, “Predefined Contents” were the contents prepared by the research group based on the needs assessment of clinical nurses before the training program began (see results in Table S2). In contrast, “Emergent Resources” were program contents which emerged from the training process (Fig. 2). The Predefined Contents of this training program; including learning goals, performance objectives, assignments, and learning materials (videos, handouts, and literature); were designed based on the task inventory of every unit, nursing research textbooks, and pedagogical theories and methods [21, 23]. The training process model (Fig. S3 in Additional File 1) was designed based on the framework of blended emergent teaching (Fig. 2) to guide the implementation of the blended emergent training program. The planned training schedule of the training program (Table S4 in Additional file 1) was designed based on the complexity of the unit contents, the results of the nominal group technique session, and the training process model.

### Development

An online learning environment (i.e., an interactive online platform) was developed. Unit assignments, learning videos, and handouts were developed, and appropriate reading materials were selected, based on the prerequisite knowledge and skills of the task inventories (Fig. S2). Furthermore, a formative evaluation questionnaire and summative evaluation instruments were selected or developed based on the purpose of this study.

### Implementation

We implemented the research training program according to the planned training schedule and training process model (see Table S4 and Fig. S3 in Additional file 1). During the training implementation process, we re-entered the other stages (A, D, D, E), as needed, to dynamically and iteratively refine the training program based on the emergence of new data (Fig. 2) from the emergent teaching processes and feedback from the formative evaluations (E).

### Evaluation

The formative evaluation is the central and ongoing “Evaluation” component of ADDIE, where the results of the ADDI components are evaluated to further refine the developing training program (See Fig. 1). For example, a research group meeting was organized during the Design and Development stages to *evaluate* the results of those stages. During the Implementation stage, the formative evaluation questionnaire was used to regularly evaluate each learner’s satisfaction and to request suggestions for improving the training contents, methods, and schedule. Meanwhile, the online interaction records, learners’ online learning processes and behaviors (monitored by the online platform), and observations from the face-to-face emergent seminars, were all analyzed as part of the formative evaluation for refining the training. The summative evaluation (reported in detail in the other publication from this project) was used at the end of the research project to evaluate the overall effects of the blended emergent research training program for clinical nurses.

## Results

### Analysis

Learners involved in this study were all clinical nurses, with a bachelor’s degree or master’s degree, having more than one-year of working experience, and were capable of using smartphones and laptops. Based on the results of the data collected in literature reviews and a focus group interview of clinical nurses, the common types of nursing research training programs, limitations of those programs, and corresponding solutions are listed in Table S1. All proposed solutions were considered during the development of the training program. Based on the corresponding solutions, a blended emergent research training for clinical nurses based on the needs assessment and related theoretical framework was proposed as a potentially effective way to improve the research competence of clinical nurses.

To provide theoretical guidance for the program development, a theoretical framework of blended emergent teaching was constructed based on a scoping and critical review of the literature related to emergent teaching (e.g., [19, 24–26]) and blended learning (e.g., [20, 27]). In the framework (Fig. 2), “Predefined Contents” refers to the contents predefined by the research team and developed based on the literature review and the learners’ needs (i.e., the results of the nominal technique group session). After the Predefined Contents were presented to the learners, there were various interactions among learners, learning resources, peers, and teachers [25]. During the interaction process, emergent resources (predictable and unpredictable) would emerge as the outputs of these interactions [28]. “Predictable emergence” refers to an emerging training process that was predictable. For example, after learners mastered pre-defined knowledge contents about quantitative and qualitative research, we could predict that the learners could then differentiate between these two types of studies. “Unpredictable emergence” refers to emerging processes that were harder to predict as a specific outcome. For example, after the discussion of an after-class assignment, some unexpected questions were proposed by learners. The analysis, identification, and use of valuable emergent resources (e.g., common mistakes made by learners, good examples provided by learners in the discussions, and good questions deserving in-depth discussion) are crucial components of emergent teaching [19]. Emergent resources can, in turn, be used as the predefined contents in future training programs.

Table S2 lists the training needs and priorities identified and considered during the Design stage. Learning goals are shown in Table S3. These goals were all informed by the overarching aim of training program to improve the NRC of the clinical nurse participants. Resources identified as being required for the training program were: Content Resources (e.g., textbooks, literature), Technology Resources (e.g., smartphones, laptops, interactive online platforms), Instructional Facilities (e.g., rooms), and Human Resources (e.g., video makers, professionals, training assistants).

### Design

The task inventory of every unit guided our systematic instructional design. A copy of the task inventory for every unit was also provided to all participants to support their learning. One example of the task inventory designed is shown in Fig. S2. Learning goals, performance objectives, and corresponding assignments were designed for every unit. One example is shown in Table S5 in Additional file 1. The learning materials for every unit were designed based on the task inventory of the unit. The specific training program’s process model (Fig. S3) and the planned training schedule (Table S4) were provided in Additional file 1**.**

### Development

The interactive online platform was developed for online learning, communication, and interaction. The platform included front-end and back-end management. Every learner had a unique username and password (these inputs were set by the user and were confidential) to log on to the front-end (i.e., the user interface) of the platform. Every member of the research group had a unique account to log on to the front-end display and to have access to the back-end management of the platform. The front-end of the platform included a “course” module (for learning files provided to learners), a “comments” module (for learners to post their comments on the research training and learning materials), a “questions” module (for learners to propose and discuss their questions related to the learning materials), a “notes” module (for learners to make synchronous notes while learning the “course” module), and a “community” module (that allowed for: (1) trainers to post lead-in questions and after-class assignments; (2) learners to post their responses to the questions and assignments; and (3) learners to discuss and interact with others). On the back-end of the platform, teachers could adjust the front-end display of the platform, post notices, upload learning materials, and track every learner’s learning behaviors and processes.

Learning materials (videos, handouts, and literature) were developed or selected and improved by the research group. The formative evaluation questionnaire was developed by the research group. This questionnaire was used to evaluate each learner’s satisfaction regarding the training contents, methods, and schedule of the units after each face-to-face emergent seminar in the training program. For summative evaluation, the Research Competence Scale for Clinical Nurses (RCSCN), the Chinese Version of the Critical Thinking Disposition Inventory (CTDI-CV), and a training satisfaction questionnaire were selected or developed as the instruments. Further details on these study materials are available in the evaluation paper noted previously [18].

### Implementation

The three-month training program was implemented according to the training process model, the planned training schedule (Table S4 in Additional file 1), and formative evaluation feedback. The actual training schedule (Table S6 in Additional file 1) included four types of training activities: online courses, practice modules, emergent seminars, and a simulation project. The predefined contents of the unit were uploaded into the online course for learners. After learning the online course module of the unit, the learners would respond to the unit’s open-ended assignments, propose additional questions, and discuss with trainers and peers in the online “community” module. Practice modules were completed in a room with computers and a campus internet connection to train clinical nurses to use databases and software commonly used in nursing research. Emergent seminars were used for group learning and discussion activities organized based on emergent topics. The emergent topics were identified and proposed by the research group through the review and analysis of the interaction records in the online “community”. There were valuable emergent resources emerging from the interactions in the training. For example, we identified common mistakes made by learners and good examples and questions related to nursing practice and research. Trainers identified and used these emergent resources as learning materials in future training. Finally, the incorporation of the simulation project was proposed and developed based on the learners’ feedback collected during the fourth week of the training program, when learners shared that it was “easy to forget the contents learned before and we cannot make all learned contents concrete”. A two-day simulation project at the end of the training program (Table S6 in Additional File 1 was implemented to simulate all steps of the research process, and to review the key knowledge and skills learned through the training program and make those skills concrete. The simulation project wass also an output of emergent teaching that is learner-centered and flexible.

### Evaluation

The data for formative evaluation were analyzed and were used for improving the training before and during the Implementation stages. For example, feedback provided by the users after using the trial version of online platform was used to improve the online platform before the Implementation stage. The simulation project was also proposed and developed based on feedback from the formative evaluation during the Implementation stage to improve the training. By the end of the study, the training program showed positive effects on nursing research competence and critical thinking of the clinical nurse participants. The summative evaluation has been explained in more detail in the separate evaluation research paper.

## Discussion

This paper illustrates the specific process of developing a blended emergent research training program using the ADDIE model. ADDIE is not only useful for systematic instructional design in predefined teaching; our study shows that it is also valuable for rigorous instructional design in blended emergent teaching. Firstly, ADDIE is a systematic method to develop the predefined contents of blended emergent teaching. Secondly, the ADDIE model and blended emergent teaching align well because they are both iterative [19, 20]. When unpredictable outcomes (emergence) occur in blended emergent teaching, the teacher can turn to any steps from Implementation based on the needs of learners. Furthermore, formative evaluation in the ADDIE model also adheres to the dynamic and learner-centered characteristics of emergent teaching, as emergent teaching emphasizes the learners’ needs and formative evaluation could reflect the learners’ needs dynamically [19, 20].

This paper has positive implications for educators who want to use the ADDIE model to design training programs, especially blended emergent education programs. Still, as ADDIE is a macro-instructional design model, educators should consider the specific contexts of their education programs while implementing the model. In this study, we effectively identified learners’ common needs and priorities for the specific context of our study by using nominal group technique. This technique is an effective method for obtaining group needs and generating priority information. Nominal group technique has been successfully used in other pedagogical research to identify the requirements and priorities of learners and teachers [29, 30]. This paper also illustrates how to develop a training program based on an initial needs assessment and a related theoretical framework – both of which are critical for the development of well-founded, systematic, and effective training programs.

Emergent teaching is the core idea of the training program in this study. Emergent teaching is the outcome of critical reflection on the process, as well as on the outcomes, of predefined teaching. Predefined teaching is the teacher-centered teaching method commonly used in traditional education programs in which all the contents are constructed by teachers according to the teachers’ own ideas and experiences; i.e., the teaching process is pre-designed and fixed [24]. Compared to predefined teaching, which focuses mostly on performance objectives, emergent teaching focuses more on the learning process. One primary purpose of emergent teaching is to improve the student’s learning ability using personalized learning strategies [25]. Blended learning is the blend of online learning and face-to-face learning [20]. The flexibility of the blended learning method allows for the personalization of the educational material according to students’ needs that emergent teaching calls for.

A theoretical framework of blended emergent teaching (Fig. 2) was developed based on the literature closely related to emergent teaching and blended learning [19, 20, 24–26]. In blended emergent teaching, the full use of emergent resources is an iterative process that may help learners to cultivate habits of inquiry, meet students’ learning needs [31], and respond to their questions more effectively, enabling students to master new knowledge and skills. This iterative process also embodies the characteristics of learner-centeredness, dynamic processes, and creativity of emergent teaching [24].

In our training program, the face-to-face and online learning enabled by the blended learning process created an environment conducive to emergent teaching. In emergent teaching, face-to-face learning provided opportunities for learners and educators to build close relationships and effectively communicate face-to-face. These opportunities were crucial for positive interactions to give rise to emergence [20]. However, online learning provided the learners with resources, space and time to learn prerequisite knowledge and skills needed for better interactions and emergence. Furthermore, the interactions in online learning were recorded and shown as texts. With blended learning, educators have more time to analyze the texts to identify, organize, and use valuable emergent resources better. In contrast, face-to-face learning places higher demands on educators’ abilities to identify valuable emergent resources, as the interactions in face-to-face learning happen quickly in oral conversations. By combining online and face-to-face learning, the blended learning training program reaped the benefits of both forms of education. These benefits are evidenced by the positive effects in the summative evaluation of the training program, supporting the practicability and rationality of the theoretical framework.

The theoretical framework of blended emergent teaching (Fig. 2) proposed in this study can contribute to the development of theory related to blended emergent teaching. It provides a theoretical framework not only for our intervention, but also for the future development of other blended emergent education programs. Considering that most work on emergent teaching before this study was limited to theoretical discussions, this framework could support more empirical intervention studies on blended emergent teaching, providing stronger evidence to evaluate the theoretical framework and promote the development of related theories.

Furthermore, the blended emergent research training program for clinical nurses developed in this study could also be used in the future as an effective research training program for other clinical nurses. Compared to nursing research lectures, workshops, journal clubs, and short-term training programs (which are more valuable for clinical nurses who are already highly experienced in nursing research [5, 15]), the proposed training program is more suitable for clinical nurses with more limited research experience. Our program could meet such nurses’ needs better, as it includes comprehensive and multilevel contents of nursing research and is developed based on learners’ needs. Compared to the one-year traditional university research courses which include face-to-face classes, lectures, and reading materials [14], our flexible training program is more suitable for clinical nurses as they can learn the prerequisite research knowledge and skills online and asynchronously. Furthermore, the cases selected in the training program are closely related to clinical nursing, so the cases may be easier to understand for clinical nurses than broader research training programs where the cases may not be specific to clinical nursing research.

In this training program, the use of emergent resources created by learners was then used to help these learners master research knowledge and skills better. By using their own materials, learners could see how they personally contributed to the training program; such contributions can promote learner engagement [24]. In the summative evaluation, the blended emergent research training for clinical nurses showed positive effects on not only NRC, but also on the critical thinking skills of clinical nurses. However, more studies are needed to further substantiate the benefits of this blended emergent research training program for nursing education and clinical nursing practice.

### Strengths and limitations

This training program was developed following the ADDIE (Analysis, Design, Development, Implementation, Evaluation) model. It was developed based on the limitations of current nursing research training programs, the needs of clinical nurses, and the theoretical foundation of blended emergent teaching, which may address learners’ needs better than traditional forms of teaching. This study was conducted in a specific context (e.g., the participants are clinical nurses holding a bachelor’s degree or a master’s degree from a tertiary hospital). Therefore, the specific conditions of other contexts should be considered when using this training program in other contexts.

## Conclusion

The ADDIE model is appropriate and effective to guide the development of a blended emergent training program. Blended emergent teaching, with its flexible and learner-centered approach, shows potential for improving students’ learning initiative and learning outcomes. However, related research on blended emergent teaching is still deficient in the field of nursing education. Therefore, blended emergent teaching should be further explored, studied, and adopted in nursing education.

The theoretical framework of blended emergent teaching constructed in this study could be used to guide the development of other blended emergent education programs. Furthermore, this study developed a complex yet practical and well-structured blended emergent research training program, based on an initial needs assessment, iterative literature search and review, and a relevant theoretical framework. This program could be used in other nursing education settings for clinical nurses to improve their research competence. By improving their NRC, individual nurses will be further supported in contributing to the advancement of evidence-based practice and nursing knowledge.

## Supplementary Information

**Additional file 1: Table S1.** Common types of current nursing research training programs, limitations of these programs, and potential solutions for addressing these limitations. **Table S2.** Training content needs and priorities resulting from the nominal group technique session. **Table S3.** Purpose statement, learning goals and corresponding units of the training program. **Table S4.** Planned training schedule. **Table S5.** Example of the learning goal, performance objectives, and assignments for every unit (Unit 2: Quantitative and Qualitative Research in Nursing). **Table S6.** Actual training schedule. **Figure S1.** Procedures for the nominal group technique session. **Figure S2.** Example of a task inventory (Unit 2: Quantitative and Qualitative Research in Nursing). **Figure S3.** Training process model for every unit.

## Data Availability

The dataset used in the current study can be made available upon reasonable request to the corresponding author.

## Abbreviation

ADDIE: Analysis, Design, Development, Implementation, Evaluation
