# Supplementary material for: Development of a blended emergent research training program for clinical nurses (part 1)
Source: BMC Nurs. 2022 Jan 4;21:2. doi: 10.1186/s12912-021-00786-x (PMC8725519; doi:10.1186/s12912-021-00786-x)
Supplement: Supplementary file 1 — Additional file 1: Table S1. Common types of current nursing research training programs, limitations of these programs, and potential solutions for addressing these limitations. Table S2. Training content needs and priorities resulting from the nominal group technique session. Table S3. Purpose statement, learning goals and corresponding units of the training program. Table S4. Planned training schedule. Table S5. Example of the learning goal, performance objectives, and assignments for every unit (Unit 2: Quantitative and Qualitative Research in Nursing). Table S6. Actual training schedule. Figure S1. Procedures for the nominal group technique session. Figure S2. Example of a task inventory (Unit 2: Quantitative and Qualitative Research in Nursing). Figure S3. Training process model for every unit. [file 12912_2021_786_MOESM1_ESM.pdf]

Table S1. Common types of current nursing research training programs, limitations of these programs, and potential solutions for addressing these limitations

**Common types of current nursing research training programs**	
Lectures, conferences, workshops, short-term training programs, and journal clubs	
**Limitations**	**Solutions**
Unmet needs in the training contents	Construct the training program contents based on a needs assessment of learners.<br>Use cases closely related to clinical nursing in learning materials and assignments.<br>Focus more on the practical research knowledge and skills often used by clinical nursing research.<br>Use emergent teaching* in training program to meet learners' needs on training contents.
Lack of systematicity	Construct and organize the training program contents systematically.
Lack of "hand-on" practical courses	Include "hand-on" practice courses of commonly used nursing research software in the training program.<br>Include practice assignments in the training program.
Hard to understand	Provide suitable media resources for better understanding.<br>Use cases closely related to clinical nursing.<br>Sequence training contents from easy to difficult.
Lack of up-to-date contents	Develop learning materials based on not only textbooks, but also on the most recent academic literature.
Teacher-centered teaching method	Construct the training program contents based on a needs assessment of learners.<br>Use emergent teaching* in the training program to implement learner-centered learning.
Lack of continuous supports	Provide continuous supports (e.g. access to learning materials and help from the trainers) after the training program.
Lack of system for feedback	Continuously collect feedback during the training process to improve the training program.
Time-limited	Use blended learning**.<br>Provide a longer time for the training program.
Lack of theoretical foundation	Construct the training program based on a relevant theoretical foundation.

Note: *Emergent teaching: the open, dynamic, and learner-centered teaching process.

** Blended learning: the blend of online learning and face-to-face learning.

The results above are based on the data from a literature review and a focus group interview. The focus group interview included 10 clinical nurses with a bachelor's degree or master's degree, having more than one-year of working experience, and the experience of participating nursing research training programs.

Table S2. Training content needs and priorities resulting from the nominal group technique session.

Items on the First Level	Total Score	Rank
1. Overview of Nursing Research and Research Ethics	0	11
2. Formulating Research Questions	24	2
3. Literature Searching and Management	18	4
4. Research Design	24	2
5. Research Sampling	11	7
6. Data Collection	12	6
7. Data Preparation for Analysis	3	9
8. Data Analysis	18	4
9. Academic Paper Writing	32	1
10. Research Proposal and Report Writing	6	8
11. Research outcomes dissemination*	2	10
**Items on the Second level**		
1. Overview of Nursing Research and Research Ethics		
(1) Overview of nursing research	5	
(2) Ethics in nursing research and academic integrity	0	
(3) Introduction and steps of quantitative research	18	
(4) Introduction and steps of qualitative research	10	
2. Formulating Research Questions		
(1) Key concepts and steps of research question formulation	28	
(2) Research question formulation	117	1
(3) Problem Identification*	20	
3. Literature Searching and Management		
(1) Academic search engines and Chinese and English databases commonly used in nursing research	22	
(2) Searching methods, approaches, and steps	84	2
(3) The introduction and use of literature management software	60	5
(4) English literature reading*	9	
(5) Literature reading tips*	15	
4. Research Design		
(1) Experimental study - Intervention study	27	
(2) Quasi-experimental study - Intervention study	12	
(3) Non-experimental study - Observational study	11	
(4) Rigor and validity in nursing research	20	
5. Research Sampling		
(1) Inclusion and exclusion criteria for research participants	54	8
(2) Sampling and allocation	41	11
6. Data Collection		
(1) Research variables and selection of measurement methods	81	3
(2) Biophysiological measures	19	
(3) Self-reports (questionnaire) method	67	4
(4) Interview method	12	

(5) Observation method	9	
(6) Quality control in data collection*	29	15
7. Data Preparation for Analysis		
(1) Data types in statistics	13	
(2) Coding, entry, cleaning of data	35	13
8. Data Analysis		
(1) The introduction of the windows and functions of SPSS	59	6
(2) Key concepts in data analysis	18	
(3) Descriptive analysis	14	
(4) *t*-test	11	
(5) Analysis of variance (ANOVA)	4	
(6) Chi-square test	14	
(7) Nonparametric test	10	
(8) Correlation analysis	11	
(9) Multiple linear regression analysis	12	
(10) Logistic regression analysis	7	
(11) The selection of appropriate statistical method*	21	
9. Academic Paper Writing		
(1) Title and the principles of authorship	25	
(2) Abstract and keywords	27	
(3) Introduction and background/literature	7	
(4) Method	58	7
(5) Results	43	9
(6) Discussion	43	9
(7) References	2	
(8) Evaluation of academic papers	37	12
(9) Journal selection of paper submission*	19	
(10) Steps of paper submission to journals*	35	13
(11) Tips for paper submission*	5	
10. Research Proposal and Report Writing		
(1) Research proposal writing	26	
(2) Research report writing	16	
(3) Application for ethical approval*	16	
(4) The organization of the research group*	4	
(5) Funding application*	4	
11. Research outcomes dissemination*		
(1) Patent application*	5	
(2) Posters and oral presentations for conferences*	4	

Note: We recruited the 25 participants from 15 units in a tertiary hospital (The participants of the nominal group technique session and the participants of the focus group interview were totally different). The inclusion criteria of participants were: (1) clinical nurses providing direct care to patients; (2) have the working experience of at least one year; (3) with a bachelor’s degree or master’s degree; (4) have some experience in clinical nursing research.

Characteristics of the 25 participants are: (1) age (32.40±5.39); (2) years of experience working as a nurse (10.88±6.77); (3) educational degree (bachelor's: 88%, master's: 12%).

The nominal group technique session followed these steps (Harvey and Holmes, 2012):
(1) Introduction and Explanation: Explain the objectives and process of the session. **(2) Silent Generation of Ideas:** Twenty-five clinical nurses were divided into four small groups (in four different rooms with a researcher as an organizer in each room) and asked to write down the items of needs for nursing research training contents. **(3) Sharing Ideas-Round Robin:** Participants were asked to share their own items in the small group. All the items shared were written down on the whiteboard and sorted. **(4) Group Discussion:** The predefined table (including items without "*" in Table 2), which was developed in advance by our research group based on the literature review, including first level and second level items of training topics, was provided to all participants. Participants in each small group were asked to discuss which items should be included in the training content needs table based on their shared items and the predefined table items. Based on the training content needs tables of the four small groups, all the predefined table items were put into the final training content needs table. The items (labelled with "*" in Table 2), generated from the Silent Generation of Ideas and not included in the predefined table, were sorted and added into the final training content needs table by four organizers together. **(5) Voting and Ranking:** All participants were present in a big room and shown the final training content needs table. They were then were asked to select three items from the first level items scoring from "3" to "1", and ten items from the secondary level items scoring from "10" to "1", according to their perceived importance of each item. We calculated the total score for each item to identify training content priorities.

Table S3. Purpose statement, learning goals and corresponding units of the training program

Purpose statement	Learning goals	Corresponding training units
To improve the research competence of clinical nurses.	1. Can make an introduction of nursing research.	Unit 1<br>Overview
	2. Can differentiate between quantitative research and qualitative research.	Unit 2<br>Quantitative and Qualitative Research in Nursing
	3. Can follow the principles of ethics and academic integrity in nursing research.	Unit 3<br>Research Ethics
	4. Can develop research questions, objectives, and hypotheses.	Unit 4<br>Formulating Research Questions
	5. Can search and manage literature needed.	Unit 5<br>Literature Searching and Management
	6. Can design research appropriately.	Unit 6<br>Research Design
	7. Can define research objects and variables.	Unit 7<br>Research Sampling and Research Variables
	8. Can collect and manage data appropriately.	Unit 8<br>Data Collection
	9. Can conduct quality control measures in nursing research.	Unit 9<br>Rigor and Validity in Nursing Research
	10. Can prepare the data before data analysis.	Unit 10<br>Data Preparation for Analysis
	11. Can analyze data based on research questions, objectives, and hypotheses.	Unit 11<br>Data Analysis
	12. Can write research proposals and applications for ethical approval.	Unit 12<br>Research Proposal and Application for Ethical Approval
	13. Can write research papers.	Unit 13<br>Academic Paper Writing
	14. Can evaluate research papers.	Unit 14<br>Academic Paper Evaluation
	15. Can disseminate research outputs.	Unit 15<br>Research Outputs Dissemination

Table S4. Planned training schedule

Type	Training Topics	Contents	Delivery Method	Time
	Training Introduction	Training schedule and management; How to use the learning resources online	Online	Week 1
Online Course 1	Unit 1 Overview	Introduction to nursing research	Online	Week 1
Online Course 2	Unit 2 Quantitative and Qualitative Research in Nursing	Paradigms, key concepts and steps in quantitative and qualitative research	Online	Week 2
Online Course 3	Unit 3 Research Ethics	Ethics in nursing research and academic integrity	Online	Week 2
Online Course 4	Unit 4 Formulating Research Questions	Research problems, research questions, and hypotheses	Online	Week 3
Online Course 5	Unit 5 Literature Searching and Management	Literature searching skills and tips; Databases commonly used in nursing research; NoteExpress (a literature management software in Chinese)	Online	Week 4
Emergent Seminar 1	Seminar on Unit 1 to Unit 4	Discussion on emergent questions and learning resources	Face-to-Face	Week 4 Sat.
Practice Module 1	*Unit 5 Literature Searching and Management*	Using at least two databases (one Chinese database and one English database) to search the related literature based on a research question, and to use NoteExpress to manage the literature searched.	Face-to-Face	Week 4 Sat.
Online Course 6	Unit 6 Research Design	Specific types of quantitative studies	Online	Week 5
Online Course 7	Unit 7 Research Sampling	Sampling; Allocation	Online	Week 6
Online Course 8	Unit 8 Data Collection	Data collection methods; How to obtain questionnaires needed	Online	Week 6
Online Course 9	Unit 9 Data Preparation for Analysis	Coding, entry, cleaning for data	Online	Week 7
Emergent Seminar 2	Seminar on Unit 6 to Unit 7	Discussion on emergent questions and learning resources	Face-to-Face	Week 7 Sat.
Emergent Seminar 3	Seminar on Unit 8 to Unit 9	Discussion on emergent questions and learning resources	Face-to-Face	Week 7 Sat.
Online Course 10	Unit 10 Data Analysis	Concepts in statistics; Descriptive statistics (SPSS); Inferential statistics (SPSS)	Online	Week 8
Online Course 11	Unit 11 Rigor and Validity in Nursing Research	Internal validity; Construct validity; External validity; Strategies to improve rigor and validity in nursing research	Online	Week 9
Online Course 12	Unit 12 Research Proposal and Application for Ethical Approval	How to write a research proposal and an application form for ethical approval	Online	Week 9
Emergent Seminar 4	Seminar on Unit 11 to Unit 12	Discussion on emergent questions and learning resources	Face-to-Face	Week 9 Sat.
Practice Module 2	*Unit 10 Data Analysis*	How to use SPSS to make simple data analysis	Face-to-Face	Week 9 Sat.
Online Course 13	Unit 13 Academic Paper Writing	Basic structure of academic papers; Writing resources and tips	Online	Week 10
Online Course 14	Unit 14 Academic Paper Evaluation	Academic paper evaluation key points; Evaluation instruments	Online	Week 10
Online Course 15	Unit 15 Research Outputs Dissemination	Academic paper submission; Conference abstract submission; Patents application	Online	Week 11
Emergent Seminar 5	Seminar on Unit 13 to Unit 15	Discussion on emergent questions and learning resources	Face-to-Face	Week 11 Sat.

Table S5. Example of the learning goal, performance objectives, and assignments for every unit (Unit 2: Quantitative and Qualitative Research in Nursing)

**Learning goal**	**Performance objectives**
1. Can differentiate between quantitative research and qualitative research	1.1 Provide research papers of different types, can correctly identify all quantitative research papers.
	1.2 Provide research papers of different types, can correctly identify all qualitative research papers.
**Assignments:**<br>1. Can you identify the quantitative research papers and qualitative research papers, respectively, from the research papers provided.<br>2. How do you differentiate between quantitative research and qualitative research?	

Table S6. Actual training schedule

**Type**	**Training Topics**	**Contents**	**Delivery Method**	**Time**
	Training Introduction	Training schedule and management; How to use the learning resources online	Online	Week 1
Online Course 1	Unit 1 Overview	Introduction to nursing research	Online	Week 1
Online Course 2	Unit 2 Quantitative and Qualitative Research in Nursing	Paradigms, key concepts and steps in quantitative and qualitative research	Online	Week 2
Online Course 3	Unit 3 Research Ethics	Ethics in nursing research and academic integrity	Online	Week 2
Online Course 4	Unit 4 Formulating Research Questions	Research problems, research questions, and hypotheses	Online	Week 3
Online Course 5	Unit 5 Literature Searching and Management	Literature searching skills and tips; Databases commonly used in nursing research; NoteExpress (a literature management software in Chinese)	Online	Week 4
Emergent Seminar 1	Seminar on Unit 1 to Unit 4	Discussion on emergent questions and learning resources	Face-to-Face	Week 4 Sat.
Practice Module 1	*Unit 5 Literature Searching and Management*	Using at least two databases (one Chinese database and one English database) to search the related literature based on a research question, and to use NoteExpress to manage the literature searched.	Face-to-Face	Week 4 Sat.
Online Course 6	Unit 6 Research Design<br>Rigor and Validity in Nursing Research*	Specific types of quantitative studies; Internal validity; research design-related contents in Unit 11 Rigor and Validity in Nursing Research*	Online	Week 5
Online Course 7	Unit 7 Research Sampling	Sampling; Allocation	Online	Week 6
Online Course 8	Unit 8 Data Collection<br>Research Variables*	The identification of research variables*; Data collection methods; How to obtain questionnaires needed	Online	Week 6

Online Course 9	Unit 9 Data Preparation for Analysis	Coding, entry, cleaning for data	Online	Week 7
Emergent Seminar 2	Seminar on Unit 6 to Unit 7	Discussion on emergent questions and learning resources	Face-to-Face	Week 7 Sat.
Emergent Seminar 3	Seminar on Unit 8 to Unit 9	Discussion on emergent questions and learning resources	Face-to-Face	Week 7 Sat.
Online Course 10	Unit 10 Data Analysis	Concepts in statistics; Descriptive statistics (SPSS); Inferential statistics (SPSS)	Online	Week 8
*Practice Module 2**	*Unit 8 Data Collection**<br>*Unit 9 Data Preparation for Analysis**	Using WJX to make an e-questionnaire*(WJX is a free and user-friendly e-questionnaire maker commonly used in China.); Using SPSS or EpiData Entry to prepare data for analysis*	Face-to-Face	Week 8 Sat.
Online Course 11	Unit 11 Rigor and Validity in Nursing Research	Internal validity; Construct validity; External validity; Strategies to improve rigor and validity in nursing research	Online	Week 9
Online Course 12	Unit 12 Research Proposal and Application for Ethical Approval	How to write a research proposal and an application form for ethical approval	Online	Week 9
Emergent Seminar 4	Seminar on Unit 11 to Unit 12	Discussion on emergent questions and learning resources	Face-to-Face	Week 9 Sat.
Practice Module 3	*Unit 10 Data Analysis*	How to use SPSS to make simple data analysis	Face-to-Face	Week 9 Sat.
Online Course 13	Unit 13 Academic Paper Writing	Basic structure of academic papers; Writing resources and tips	Online	Week 10
Online Course 14	Unit 14 Academic Paper Evaluation	Academic paper evaluation key points; Evaluation instruments	Online	Week 10
Online Course 15	Unit 15 Research Outputs Dissemination	Academic paper submission; Conference abstract submission; Patents application	Online	Week 11
Simulation Project* *(To completely experience all research processes learned over the course of the prior 11 weeks)*	Day 1*	Session 1*<br>Research question, hypotheses, and objectives<br>Research population and variables<br>Ethics<br>Literature searching plan<br>Research design<br>Research sampling<br>Data collection<br>Strategies to improve research quality	Face-to-Face	Week 11 Sat.<br>8:30-12:00
		Session 2*<br>Data preparation<br>Data analysis	Face-to-Face	Week 11 Sat.<br>13:30-18:00
	Day 2*	Session 3*<br>Research proposal writing	Face-to-Face	Week 11 Sun.<br>8:30-12:00
		Session 4*<br>Research paper writing (make an outline of the research paper)<br>Paper evaluation	Face-to-Face	Week 11 Sun.<br>13:30-18:00

Note: *These components were added during the I(implement) phase based on learners' needs emerging in the blended emergent teaching.

Figure S1. Procedures of the nominal group technique session

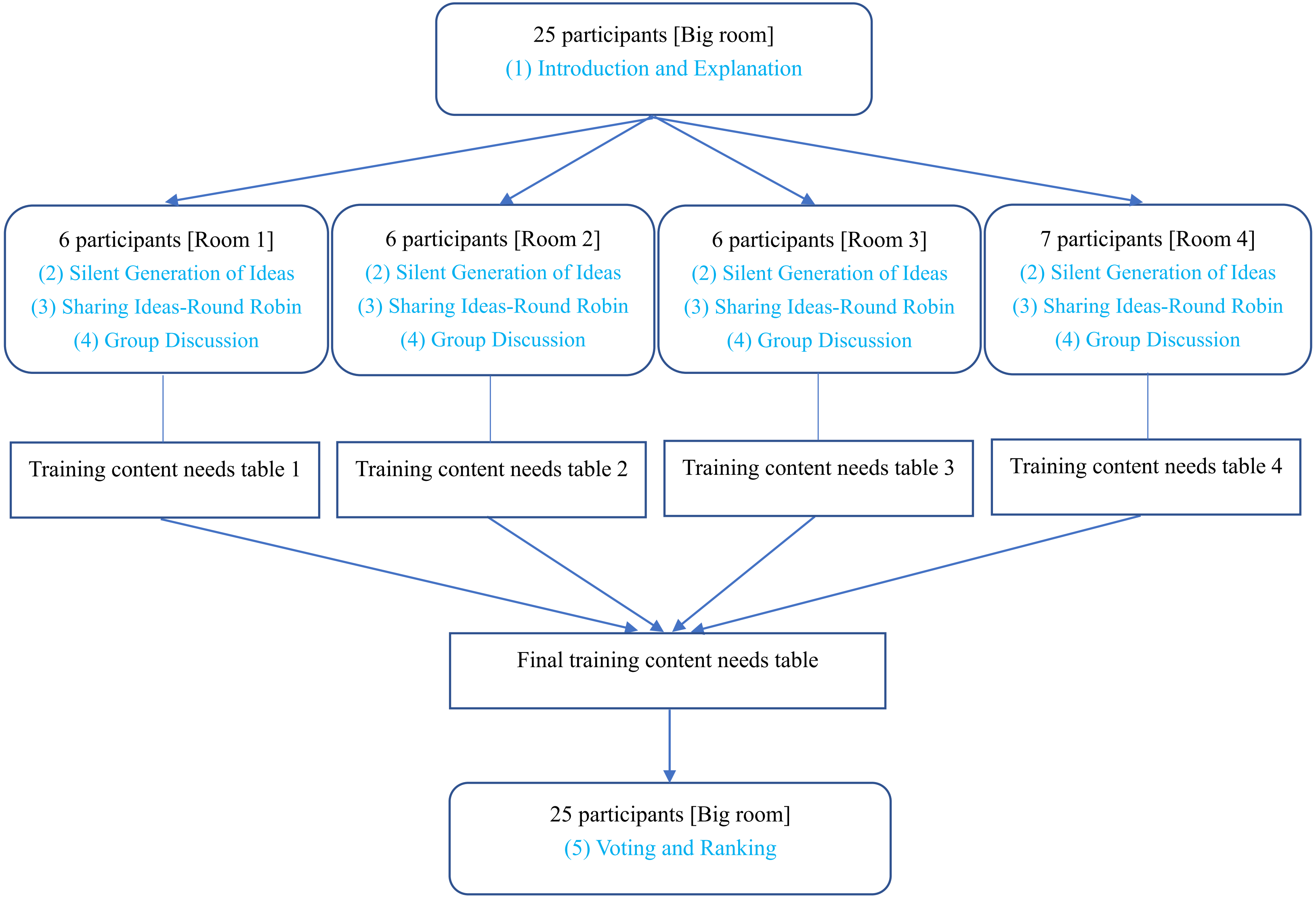

Figure S2. Example of a task inventory (Unit 2: Quantitative and Qualitative Research in Nursing)

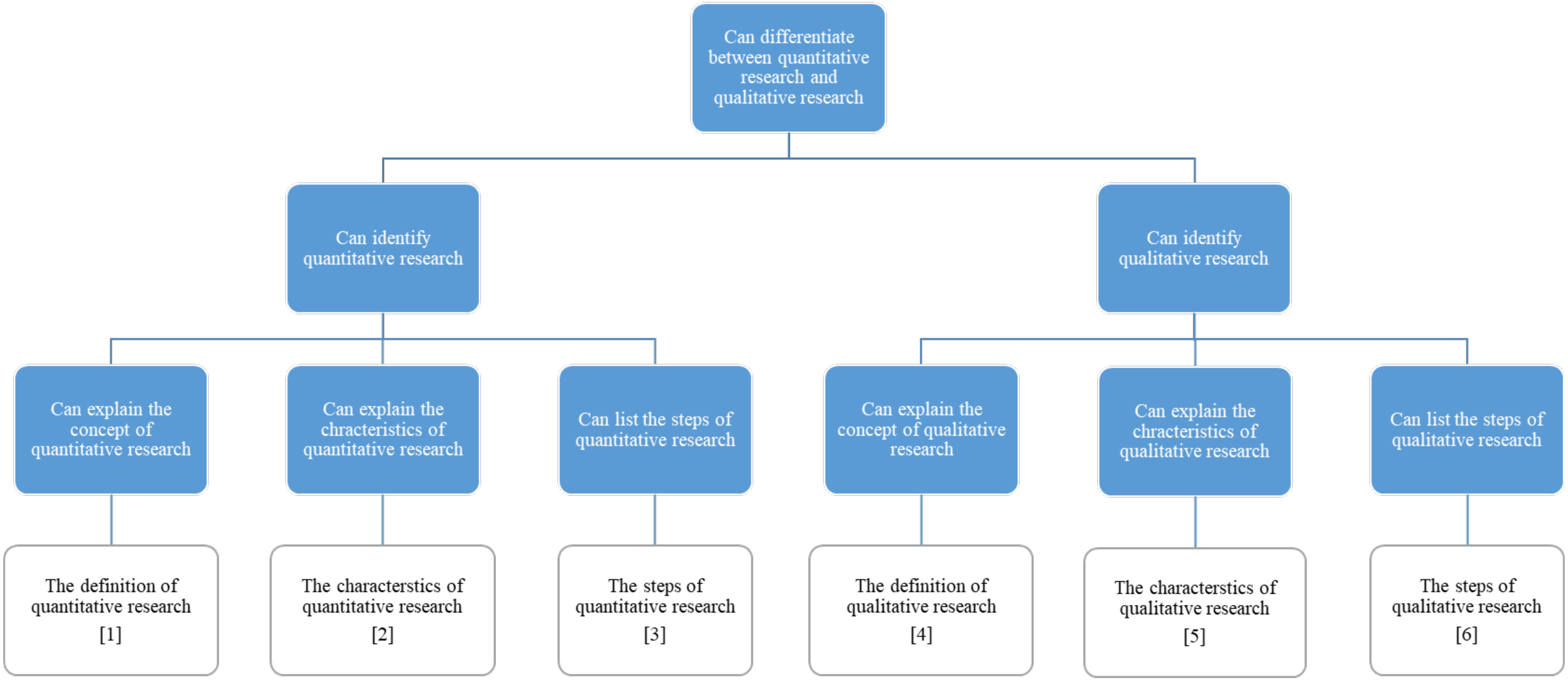

Note: The block on the top level is the learning goal of the unit. The blocks on the middle levels are the performance tasks required by the learner to achieve a learning goal. The white blocks on the bottom level are the prerequisite knowledge and skills needed for performance tasks. The [numbers] are used to match the contents in the white blocks with the learning contents in the handout of the unit, to help learners find the corresponding contents in handout quickly.

Figure S3. Training process model for every unit

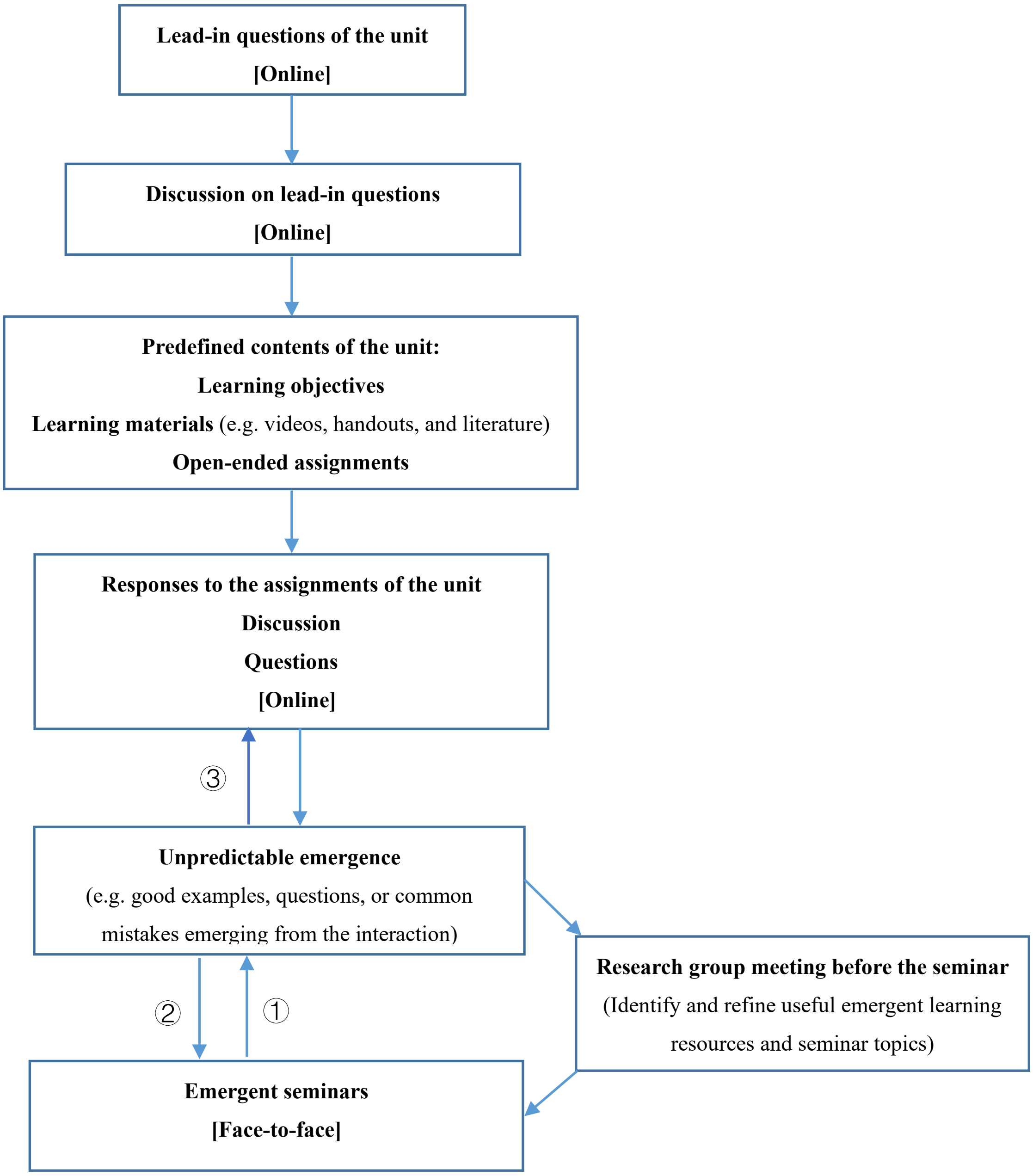

Note:

Lead-in questions of the unit were uploaded in the "community" module online one week before the **predefined contents of the unit** were uploaded. After clinical nurses discussed on the lead-in questions and shared their opinions, predefined contents would be uploaded online. Open-ended assignments (i.e., open-ended questions or the tasks which asked clinical nurses complete research activities based on their own research questions or personal experience) would then be uploaded into the community. Next, the clinical nurses would post their **responses** to the assignments and **discuss** with others participating in the community). They could also propose other **questions** in the community for discussion. This plan allowed for the spontaneous **unpredictable emergence** of new training materials and new ideas from the interaction online. The research group would then identify and refine useful emergent learning resources and seminar topics to plan face-to-face emergent seminars. During the **emergent seminars**, further emergent resources may also emerge from the interaction process in the emergent seminars①. Trainers would recognize and use these emergent learning resources flexibly in the seminars②. If needed, the emergent learning resources could also be further used and discussed online after the seminars③.
